# Extrusion and characterization of recycled polyethylene terephthalate (rPET) filaments compounded with chain extender and impact modifiers for material-extrusion additive manufacturing

**DOI:** 10.1038/s41598-023-41744-8

**Published:** 2023-09-25

**Authors:** Ola Rashwan, Zachary Koroneos, Trent G. Townsend, Matthew P. Caputo, Robert J. Bylone, Brennan Wodrig, Kirk Cantor

**Affiliations:** 1 Pennsylvania State University - Harrisburg, 777 W Harrisburg Pike, Middletown, PA 17057 USA; 2 Pennsylvania State University -Shenango, 147 Shenango Avenue, Sharon, PA 16146 USA; 3 Recycling Markets Center, Pennsylvania State University - Harrisburg, 777 W Harrisburg Pike, Middletown, PA 17057 USA; 4 Plastics Innovation and Resource Center, Pennsylvania College of Technology, One College Avenue, Williamsport, PA 17701 USA

**Keywords:** Chemistry, Materials science

## Abstract

The continuous growth of annual production and consumption of polyethylene terephthalate (PET) is coined with increasing waste that leaks into the environment, landfills and oceans as microplastics and nano plastics fragments. Upcycling the recycled PET to make a feedstock for the fast-growing material-extrusion additive manufacturing (MEX-AM) technology can contribute to the solution and supports the concept of sustainable materials. In this work, extrudable filaments comprising recycled polyethylene terephthalate (rPET) with low-cost additives, such as pyromellitic dianhydride (PMDA) as a chain extender, styrene-ethylene-butylene-styrene terpolymer functionalized with maleic anhydride (SEBS-g-MA), a thermal modifier and toughening agent, ethylene-ethyl acrylate-glycidyl methacrylate terpolymer (E-EA-GMA), a functional reactive elastomeric impact modifier and ethylene-ethyl-acrylate (EEA), a non-reactive elastomeric impact modifier, have been fabricated using the twin-screw extruder. The optimum extrusion process parameters for producing uniform filaments of different rPET compounded formulations have been identified, this includes the extrusion die temperature of 280 °C and the screw speed of 150 ± 3 rpm. The compounded filaments are then printed into standard ASTM test specimens for thermal characterization and mechanical characterization, including glass transition and melting temperatures, crystallinity and crystallization temperature, tensile strength, tensile modulus, ductility, flexural strength, and Izod impact energy. Furthermore, the melt flow index for the filaments was measured. More significantly, the experimental data showed that compounding rPET with such additives in the reactive twin-screw extrusion process results in uniform filaments that display advantageous thermal and mechanical properties and can be used as a feedstock in the MEX-AM technology. This study suggests that compounding the recycled PET pellets with low-cost additives while extruding them into filaments for MEX-AM offers excellent potential to make high-value-added customized products from a sustainable polymer feedstock, such as prototyping, tooling, testing components or end-use internal components for small machines and cars.

## Introduction

Polyethylene terephthalate (PET) is one of the most widely used thermoplastics. It is primarily used in food packaging, including beverage bottles, trays, containers, straps, and as fibers in the textile industry^1^. PET is a semi-crystalline thermoplastic that belongs to the polyester family; it is recyclable, strong, and lightweight, making it an ideal material in the rigid packaging industry^2^. The global polyethylene terephthalate (PET) market has grown exponentially in recent decades^3^. The main concerns of PET products are their short- life cycle and high- consumption. Recycling PET has been an inevitable mitigation strategy to combat PET’s high consumption and waste^4,5^.

Three recycling methods are being used with PET: mechanical, chemical and incineration^6–8^. The most common method used in recycling PET is mechanical recycling due to its simplicity, low cost, and low energy consumption, which produces less greenhouse gas that contributes to the global warming crisis^9^. However, the significant shortcomings of mechanical recycling are thermomechanical and hydrolytic degradations that result from a chain scission, a molecular weight reduction, and a decrease in intrinsic viscosity^10–13^ One common approach to resolve the degradation concerns and improve the inherent viscosity and properties was to compound the rPET pellets with additives when processing them into finished products. Schiers and Long^14^ suggested many additives and formulations that could improve the properties of PET and overcome its deficient properties, such as hygroscopicity, slow crystallization, oxidation and loss of intrinsic viscosity during extrusion and brittle fracture.

Several researchers have investigated the effect of compounding virgin and recycled PET with different chain extenders to produce engineering-grade PET^14–29^. Ample evidence in the literature showed the effectiveness of using pyromellitic dianhydride (PMDA). In the reactive extrusion, the PMDA reacts with the PET end groups -hydroxyl and carboxylic acid groups- and causes chain extension and branching of PET. It was noted that the primary use of PMDA was to increase the melt strength and allow PET to foam when added from 0.05 to 2.0 wt%^14,18^. Other advantages include increasing the intrinsic viscosity and tensile strength and making PET more suitable for blow molding, injection molding and extrusion processes.

Awaja et al.^28,29^ studied the effect of the reactive extrusion process’s PMDA concentration and residence time on intrinsic viscosity, crystallinity, and thermal transitions. They concluded that the concertation of the PMDA is the most critical factor in obtaining an acceptable intrinsic viscosity and a stable extrusion system that does not allow gel formation. The residence time was identified as a lesser critical. They recommended a PMDA concertation of 0.2 wt% but less than 0.3 wt%, and the residence time should be higher than 45 s. Furthermore, neither PMDA concentration nor the residence time affected the glass transition temperature (T_g_). Increasing the PMDA concentration and extrusion residence time lowered the melting and crystallization temperatures. Additionally, the crystallinity of compounded rPET / PMDA extrudates was lower than the neat rPET extrudates.

Another comprehensive study by Nascimento et al.^20^ studied blending rPET with 0.1–1.0wt% of PMDA. Similar conclusions were drawn; an increase in the viscosity, a decrease in crystallinity, and no significant change in thermal stability were observed.

More studies on the rheological behavior showed that adding PMDA increased the rPET molecular chain length and branching through the chemical reactions with the end groups of rPET. The melt flow index and melt strength of the rPET mixed with PMDA were investigated by Wang et al.^23^ and a decrease in the melt flow index and an increase in the melt strength were noted as the weight percent of PMDA increased from 0.25 to 1.25 wt%. Hirach et al.^22^ also noted an increase in the viscosity and molecular weight when different weight fractions of PMDA ranging from 0.1 to 0.3 wt% were added to PET.

Most studies above focused on the thermal and rheological properties of the reactive extrusion of the compounded rPET/PMDA pellets for the injection molding processes; however, Qu et al.^19^ studied the spinnability, rheological, thermal, mechanical, and morphological properties of the melt-spun short fibers of virgin PET and rPET modified by PMDA. They reached a similar conclusion on the effectiveness of PMDA on processability and properties improvement.

Most PET applications, such as food packaging, strapping, and automotive components, require high-impact resistance. Thus, toughening agents and impact modifiers have been added to the virgin and recycled PET to improve impact resistance and toughness^25,30,31^. Karsli^25^ compounded the recycled PET with the commercial styrene acrylic chain extender (Joncryl®) and the impact modifier (Lotader AX8900®) simultaneously in a twin-screw co-rotating extruder to improve the mechanical strength and fracture toughness of the rPET extrudates for injection molding. It was found that 2.5% Lotader® with 1% Joncryl® increased the tensile strength and toughness of r-PET. Similar to the previous findings, the thermal transition temperatures of rPET were unaffected.

In recent years, researchers and industry have become increasingly interested in developing new feedstocks for material-extrusion additive manufacturing technology (MEX-AM). The global 3D printing filament market generated $693.1 million in 2019 and is expected to increase to $7.08 billion by 2030. Developing sustainable rPET filaments to embrace the circular economy concept has great potential in the fast-growing additive technology^32^. Click or tap here to enter text.According to the United States EPA (Environmental Protection Agency) definition of circular economy, “a circular economy reduces material use, redesigns materials, products, and services to be less resource intensive, and recaptures “waste” as a resource to manufacture new materials and products”^33^. Reuse and integration of recycled polymers in the fast-growing MEX -AM technology is anticipated to have tangible impacts on sustainable/green manufacturing and reinforce the circular economy paradigm^34^.

Few researchers have investigated using rPET to make lab-scale filaments for MEX-AM technology^35–38^. They used single-screw and twin-screw extrusion to fabricate the filaments, and they investigated the thermal, rheological, and mechanical properties of the filaments and printed samples; their studies concluded that recycled PET filament could replace the current commercial filaments, such as acrylonitrile butadiene styrene (ABS). To our knowledge, no study has examined compounding rPET pellets with additives and extruding them into uniform sustainable filaments for the fast-growing MEX-AM industry.

Using rPET filaments to make high-value-added customized products with a longer life cycle using material-extrusion additive manufacturing technology (MEX-AM) could have a pronounced environmental impact. Since the proposed concept of making 3D printing filament of recycled PET is new, there is no life cycle assessment (LCA) studies have been published yet to the best of the authors’ knowledge; however, there are numerous studies have been published on the LCA of recycled PET for different applications; these studies have proved that the recycled polymers consume less energy by 84% and having less greenhouse gas emission by 74%^39^. We anticipate that the recycled PET filament for use in additive manufacturing offers a promising avenue on several fronts of sustainability including environmental impacts, economic, and energy consumption. Recycled PET filament can be cost-effective compared to neat PET filament due to the lower cost of sourcing recycled PET materials. The recycling process reduces the need for extracting and refining raw materials, resulting in lower material costs, which in turn reduces energy consumption. The environmental impact of recycled PET filament also could have potential benefits by reducing emissions, water usage, and overall carbon footprint.

In this study, rPET pellets were compounded with a chain extender, a toughening agent, and impact modifiers and extruded into AM filaments. The mechanical properties of the printed parts were anticipated to improve, so long-term/high-value-added customized products could be made. The aim of this study was twofold: 1. to evaluate the possibility of the twin-screw extrusion of the recycled PET pellets compounded with a low-cost chain extender and impact modifiers into filaments that could be printable. 2. to shed some light on the thermal behavior and the mechanical properties of the compounded filaments and printed samples.

The following sections present the extrusion of mechanically recycled PET pellets with the chain extender, toughening agent, and impact modifiers. The filaments and printed parts’ rheological, thermal, and mechanical properties were tested and analyzed. Furthermore, the fractured surfaces of the tensile test samples were examined.

## Materials and methods

The effect of the chain extender, toughening agent, and impact modifiers on the extrusion of the rPET 3D printing filament, the thermal behavior and the mechanical properties of the printed samples have been investigated in this project. This section discusses the preparation of the rPET pellets and additives, the extrusion parameters, and the printing parameters of the ASTM (American Society for Testing and Materials) tests’ samples, including the mechanical and thermal tests.

### Materials

Mechanically recycled PET (rPET) pellets were acquired from Cougle’s Recycling Inc., Pennsylvania, USA, through the Recycling Markets Center at Penn State Harrisburg. The data sheet provided by the rPET pellets’ supplier listed the following properties of the rPET pellets: intrinsic viscosity of 0.8 ± 0.02 dl/g, a melting point of 247 ± 2 °C, moisture content of a max of 0.1 wt%, a weight of 100 pcs (pellets) is 1.6 ± 0.2 g, and fine particles of a max of 100 ppm. The following four additives were obtained for compounding with rPET pellets: (1) Pyromellitic dianhydride (PMDA), a chain extender (Sigma Aldrich), (2) Styrene-ethylene-butylene-styrene terpolymer functionalized with maleic anhydride (SEBS-g-MA), a thermal modifier and toughening agent (FG1901X, KRATON Polymers), (3) Ethylene-ethyl acrylate-glycidyl methacrylate terpolymer (E-EA-GMA), a functional reactive elastomeric impact modifier (Lotader AX 8900, Palmer Holland), and (4) Ethylene- ethyl- acrylate (EEA), a non-reactive elastomeric impact modifier (Sigma Aldrich). All additives were used as purchased without modification.

### Extrusion of the 3D printing filament

The first rPET blend consists of rPET and PMDA (rPET/PMDA). A 0.3 wt% of PMDA was bag mixed with rPET pellets for 3 min and poured into the extruder feeder. The second blend contains a blend of 0.3 wt% PMDA, 5.0 wt% SEBS-g-MA, 1.5 wt% E-EA-GMA, and 3.6 wt% of EEA. The 30:70 ratio of functional reactive and non-reactive impact modifiers, E-EA-GMA and EEA, has been used to ensure uniform dispersion of the rubber additives into the rPET matrix^14^. The rPET pellets were dried at 120 °C for 12 h in a vacuum dryer (Macguire). The four other additives (PMDA, SEBS, E-EA-GMA, EEA) were dried in separate vacuum dryers (Thermo Precision Scientific) for 4 h at 50 °C and kept in a vacuum dryer until extrusion. In this paper, the first blend is called “rPET/PMDA”, and the second is called “rPET/IMs”.

**Figure 1:**
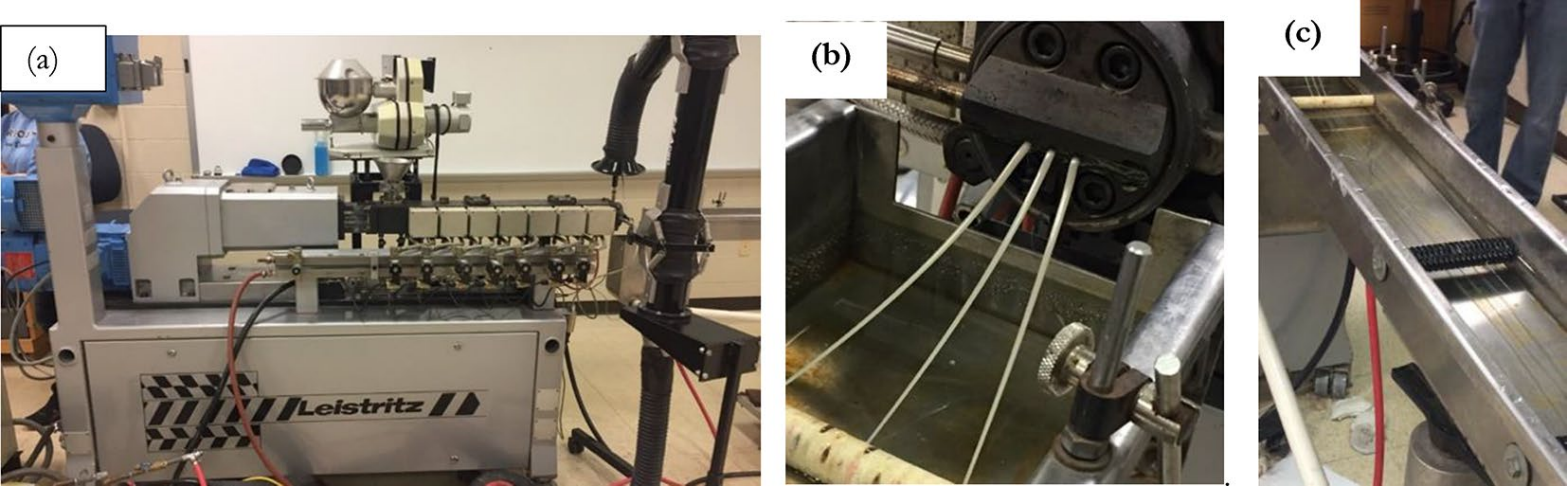
(**a**) Twin-screw extruder, (**b**) three strands simultaneously out of the die, (**c**) water cooling path.

A twin-screw co-rotating extruder (Leistritz) was utilized and shown in Fig. 1a. First, the dried rPET pellets and additives were bag-mixed for 3 min; then, each compounded rPET blend was starve-fed using a volumetric feeder into the hopper of the twin- screw extruder. The throughput of the volumetric feeder was 10.4 kg/h. The speed of the screws was set at 150 ± 3 rpm throughout all extrusion processes. The extruder has eight individually-controlled temperature zones. The zones’ temperatures for each filament type: (1) rPET, (2) rPET /PMDA and (3) rPET /IMs are listed in Table 1. The extrusion of rPET/PMDA and rPET/IMs had the die temperatures increased to 280 °C to reach the reaction temperature between rPET with the additives^40^. The die of the twin-screw extruder has three output holes, producing three strands simultaneously, as shown in Fig. 1b. The filaments were water-cooled in a water bath. Within the water bath, cylindrical metal pieces were used to maintain a straight path toward the filament puller, as depicted in Fig. 1c. The filament was fed into the filament puller, which was adjusted based on the diameter measurements using a digital micrometer. The filament puller speed was set at 0.36 m/s, 0.31 m/s, and 0.28 m/s for the rPET, rPET /PMDA, and rPET /IMs, respectively. The filament diameter was measured on two axes: parallel and perpendicular to the force of the puller. Once the diameter was within 1.75 ± 0.05 mm, the filament was spooled on a mechanical spooler.

**Table 1:** Temperatures of the eight extrusion zones of the plain and compounded rPET filaments.

Compound	Zones ‘temperatures (°C)							
	1	2	3	4	5	6	7	8
rPET	220	220	240	240	250	250	250	260
rPET + PMDA	220	230	250	260	260	275	280	280
rPET + IMs	220	230	250	260	260	275	280	280

### Material extrusion-based additive manufacturing (MEX-AM)

The ASTM standards’’ specimens for tensile, 3-point flexural, and Izod tests were printed. The filaments were dried at 65° Celsius for 2–3 h before printing. The filament is then stored in a dehumidifier to mitigate the hygroscopicity of rPET. The Ultimaker Cura 5.0 slicing software was used to generate the G-codes for the Ender 5 Pro Printer (CREALITY). The ironing feature was activated, and no support has been used. The printing parameters are tabulated in Table 2.

**Table 2:** Material Extrusion-based additive manufacturing parameters.

Nozzle temperature	260 °C	Printing bed temperature	90 °C
Infill density	100%	Infill pattern	Rectilinear/lines
Printing speed	50 mm/s	Wall thickness:	1 mm
Raster angle	45/− 45°	Nozzle type	0.4 mm hardened steel
Layer thickness	0.2 mm	Top/bottom layer thickness:	0.8 mm

The printing parameters were optimized to maximize dimensional accuracy. This includes the nozzle temperature, layer height, printing speed, and printing bed temperature to avoid shrinkage during printing. The printing parameters were the same for the plain and compounded rPET filaments. The average dimensional accuracy was ± 1.5%.

### Characterization of the rPET and compounded rPET samples

In the following subsections, melt flow index measurement, thermal, mechanical, and morphological characterization have been carried out to analyze the additives’ effect on the behavior of the filaments and printed parts.

#### Melt-flow index measurement

The melt flow index was measured using Dynisco melt flow indexer according to ASMT D1238: a 6-min hold and a 10-s cut at 270 °C /2.16 kg were maintained with all samples. The test was done in triplicates, and the average melt flow index (MFI) was calculated for the neat PET and each PET blend.

#### Thermal characterization

Small pieces (approximately 5 mg) were cut from the filaments and the printed tensile specimens for the thermogravimetric analysis (TGA) and differential scanning calorimetry (DSC).

The TGA tests were conducted on an SDT Q600—TA Instruments. All samples were heated from 25 to 800 °C at 20 °C /min under a nitrogen atmosphere.

The DSC tests were performed on TA Instrument Q2000 following the ASTM D3418 standard. First, the samples were heated from room temperature to 320 °C, then cooled to − 50 °C, then heated again to 300 °C, and finally cooled to room temperature. All heating/ cooling rates were 20 °C/ min under a nitrogen flow of 50 ml/min. The second heating cycle was performed to remove the thermal history of the specimens. The data were processed using Universal analysis (UA) -TA Instruments software. The crystallinity was calculated using the following equation:

$$\% Crytallinity= \frac{{{\Delta H}_{m}-\Delta H}_{cc}}{{\Delta H}_{f}}\times 100$$

where ${\Delta H}_{m}$ is the area under the melting endotherm, $\Delta {H}_{cc}$ is the area under the exotherm cold recrystallization peak from the first heating cycle, and ${\Delta H}_{f}$ is the heat of fusion of a 100% crystalline PET of 140 J/g^41^.

#### Mechanical testing

The uniaxial tensile and 3-point flexural tests were conducted using MTS Insight 30 load frame with a 5kN load cell using the standard tensile wedges and the 3-point bending fixtures. The tensile tests were performed according to ASTM D638 on type V printed specimens and a constant displacement rate of 2 mm/min. The ultimate tensile strength and the modulus of elasticity were measured.

The 3-point flexural tests were carried out according to ASTM D790. The samples were printed with a width of 12.7 mm, a thickness of 3.2 mm and a support span was 50 mm. The displacement rate was set to 2 mm/min. The flexural strength and the strain at break were measured. A minimum of five samples were tested for each test.

IZOD impacts tests were carried out according to the ASTM 256-10 (2018) standard using tmi (testing machines inc.) impact tester. The notched samples have a square cross-section of 12.7 ± 0.2 mm, a length of 63.5 ± 2 mm, and a notch depth of 2.0 mm. The samples were tested in triplicate.

#### Fracture analysis

Fractured surfaces were cut from the failed tensile test samples using a water-cooled high-concentration diamond wafering blade (450 rpm). The fractured surfaces were air-dried and sputter coated with a gold–palladium target under an argon atmosphere for approximately 3 min with a sputtering current of 30 mA. The fractured surfaces were then examined by scanning electron microscopy using a Quanta 200 by FEI. All micrographs were captured as secondary electron (SE) images using an Everhart–Thornley detector with an acceleration voltage of 5 kV.

## Results and discussion

### Extruded rPET and compounded rPET filaments

**Figure 2:**
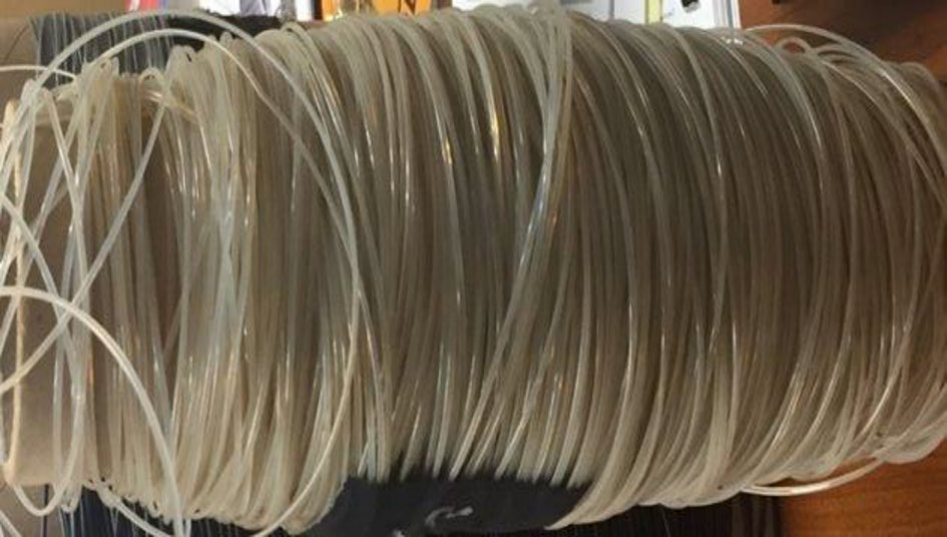
Extruded filament spool for the MEX-AM process.

The neat rPET and compounded rPET filaments of 1.75 ± 0.05 mm were extruded on the twin-screw extruder. The filaments of uniform diameters were obtained, as shown in Fig. 2. The pulling speed was adjusted to obtain the required filament diameter within the acceptable tolerances. The extrusion parameters were adjusted to obtain flexible and processable filaments during the MEX-AM. Drying the pellets before extrusion and the water cooling of the extruded filament were two crucial parameters for controlling the dimensional accuracy and flexibility of the filament.

### Melt flow index

**Figure 3:**
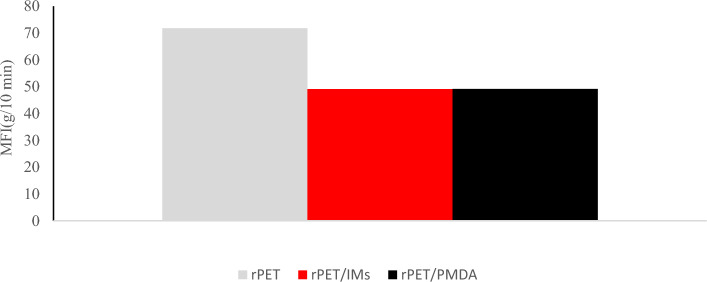
Melt flow index of rPET, rPET /PMDA and rPET/IMs filaments.

The additives generally improved the melt flow index (MFI) of rPET, as shown in Fig. 3. Adding the chain extender by itself or with the toughening agent and impact modifiers resulted in a similar MFI of 49.1 g/10 min. The improvement in MFI is attributed to the increase in the molar mass. The decrease in the melt flow index infers an increase in intrinsic viscosity and melt strength. This, in turn, allows rPET pellets to be foamed and become suitable for multiple extrusion cycles without degradation.

### Thermal analysis

**Figure 4:**
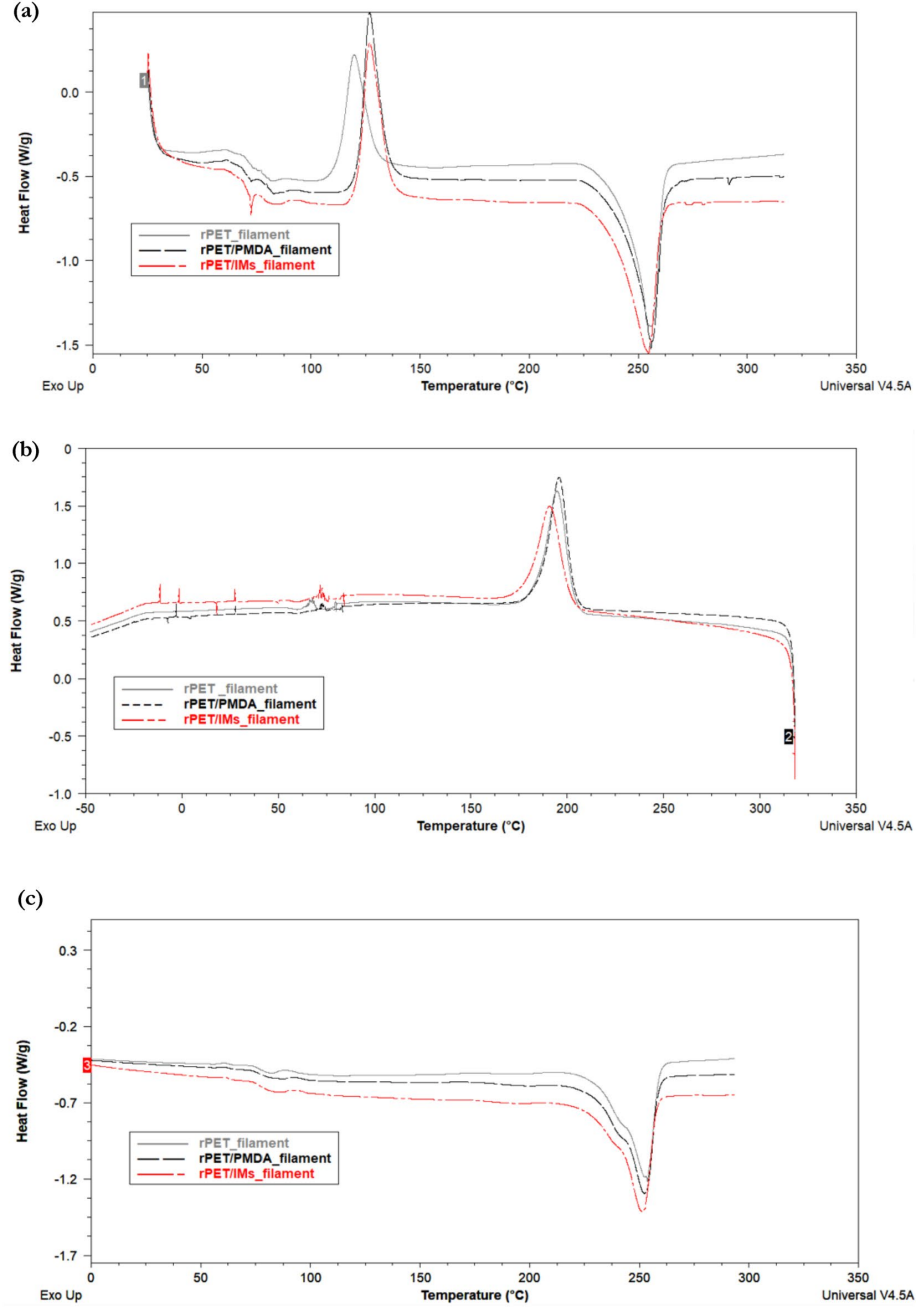
Representative differential scanning calorimetry curves for rPET, rPET/PMDA, and rPET/IMs filaments (**a**) the first heating cycle shows the recrystallization temperatures (T_cc_) and the melting peaks (**b**) the Cooling cycle shows the crystallization peaks (**c**) the second heating cycle shows the glass transition temperatures (T_g_) and the melting temperature (T_m_) after removing the thermal history.

**Figure 5:**
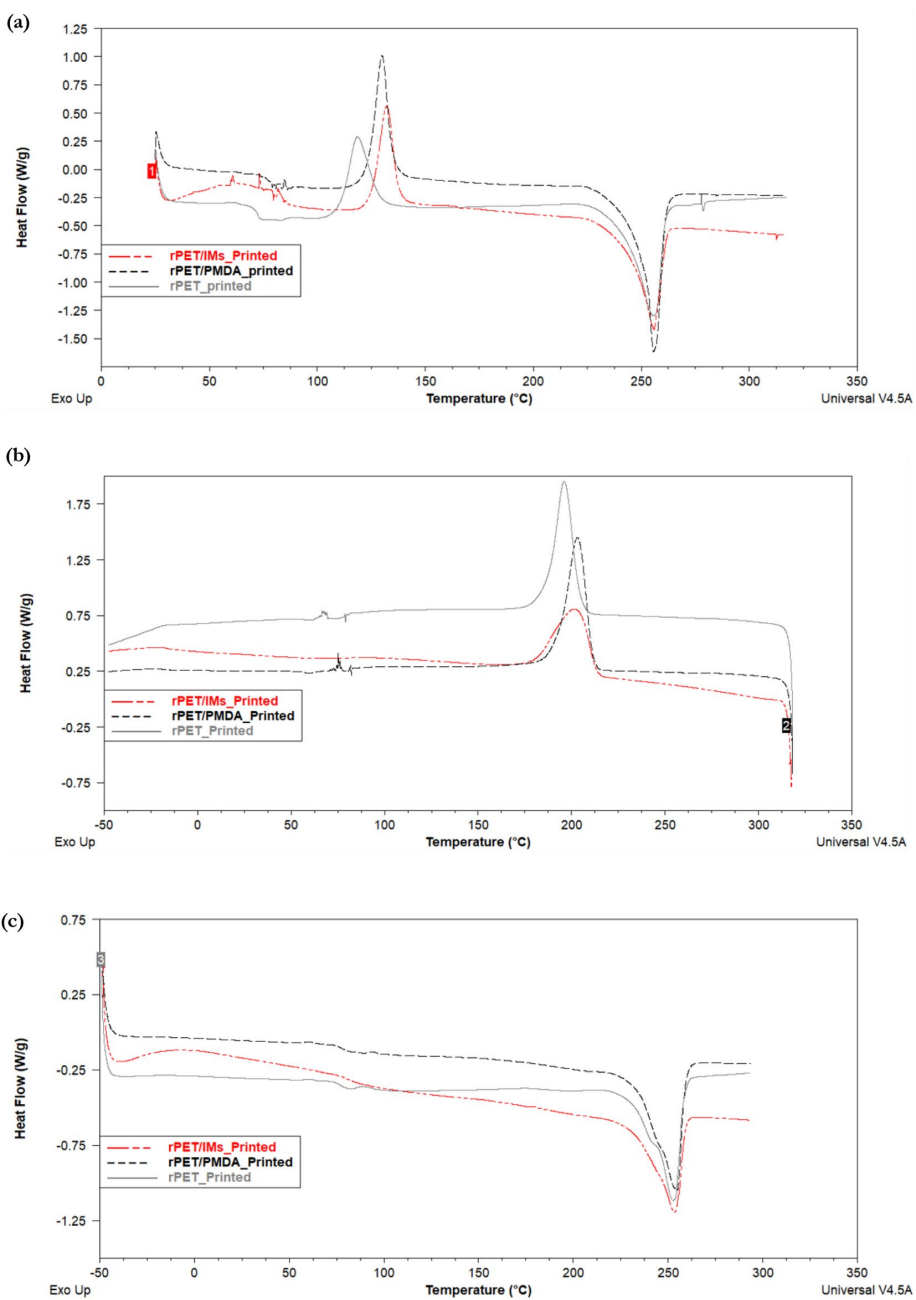
Representative differential scanning calorimetry curves for rPET, rPET/PMDA, and rPET/IMs Printed samples (**a**) the first heating cycle shows the recrystallization temperatures (T_cc_) and the melting peaks (**b**) the Cooling cycle shows the crystallization peaks (**c**) the second heating cycle shows the glass transition temperatures (T_g_) and the melting temperature (T_m_) after removing the thermal history.

The thermal behavior of the plain rPET and compounded rPET filaments and printed parts has been studied to shed some light on the effect of the additives on the glass transition temperature (T_g_), the melting temperature (T_m_), the crystallinity (%Xc) and the thermal stability. The heating and cooling curves resulting from the DSC tests are depicted in Figs. 4 and 5. Table 3 summarizes the thermal characteristics of the plain and compounded rPET filaments and printed parts. The first heating curves show the recrystallization peaks of the amorphous part of the samples and melting peaks, whereas the cooling curves show the crystallization peaks/temperatures. The curves of the second heating cycles show the glass transition temperatures and the effect of the multiple heating on the melting temperatures.

**Table 3:** The thermal properties of the plain and compounded rPET filaments.

	T_g_ [°C]	T_cc_ [°C]	T_m_,_1_ [°C]	T_m_,_2_ [°C]	T_c_ [°C]	ΔH_cc_ _[J/g]_	ΔH_m_	%Xc
rPET- filament	77.3	120.0	255.8	253.6	194.6	25.3	41.6	11.6
RPET/PMDA-filament	79.9	126.8	255.8	252.8	195.7	29.0	41.2	8.7
RPET/IM-filament	77.4	127.0	254.8	251.2	190.7	27.9	40.3	8.8
rPET- 3D printed	79.5	118.8	255.6	252.6	196.1	23.3	42.3	13.6
RPET/PMDA-3D printed	83.3	130.0	255.4	254.8	203.4	26.7	46.6	14.2
RPET/IM-3D printed	81.3	132.2	255.9	253.5	202.3	23.0	36.1	9.3

The recrystallization peaks /temperatures of the compounded rPET filaments shifted to higher temperatures due to the additives, and the peaks are larger than the plain rPET filament, indicating a higher amount of amorphous character. Similar behavior showed up in the thermograms of the 3D printed parts; the peak of the rPET/IMs shifted to a slightly higher temperature than rPET/PMDA, but the peak of rPET/PMDA is still the largest.

The crystallization peaks of the rPET/IM filament and printed sample were smaller and more broadened than those for plain rPET and rPET/ PMDA samples. They shifted to lower temperatures, indicating a decrease in the crystallization enthalpy and less crystallinity. This is likely due to the rubbery nature of the impact modifiers.

The additives slightly impact the glass transition temperatures for filaments and printed samples. The printed samples had higher average glass transition temperatures than the filament, which agreed with the trend in^14^.​ Therefore, slight adjustments to the 3D printing temperatures of the compounded rPET filaments might be needed.

The effect of the additives on the melting temperatures is not pronounced; The melting temperatures of all formulations centered around 255 °C ± 1 °C and at a slightly lower temperature of 252 °C ± 2 °C for the second heating cycle, which reveals the thermal stabilities of the compounds even after frequent melting cycles during processing and testing.

For the first heating cycles, the melting peaks were single and narrow, implying homogeneity of the rPET matrix with the additives and the uniformity of the crystallites’ sizes.

The crystallinity of the water-cooled plain rPET filament was the highest compared to the compounded filaments, and its % Xc agreed with the values reported in references^35,36^. The compounded filaments had a similar crystallinity degree. The structural disorder and the entanglements of the branched chains induced by the chain extender, toughening agent, and impact modifiers decreased the molecular mobility and diffusion into the nucleation sites resulting in small crystallites and low crystallinity. For the MEX- AM technology, an increase in crystallinity tends to increase the material’s strength and stiffness but reduces its ductility. So, the crystallinity needs to be optimized so that the filament is strong but flexible enough not to break during printing which has been achieved in this project.

The printed samples had higher crystallinity than the filaments. A similar trend was noted in reference^36^; the higher crystallinity of the printed samples might be attributed to the printing conditions, including a slow cooling rate, a high printing bed temperature, and a slow printing speed.

#### Thermal stability

**Figure 6:**
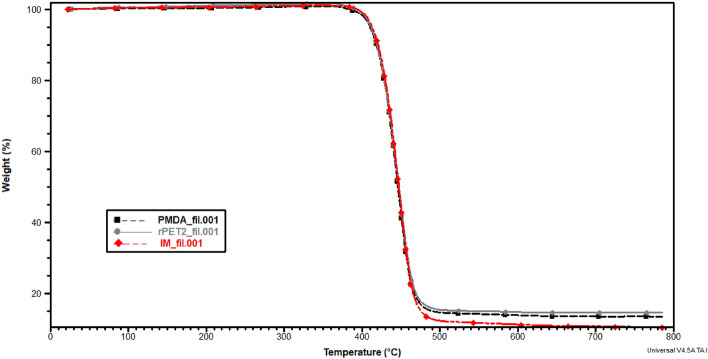
TGA curves for filament made of rPET, rPET/PMDA and rPET/ IMs.

**Figure 7:**
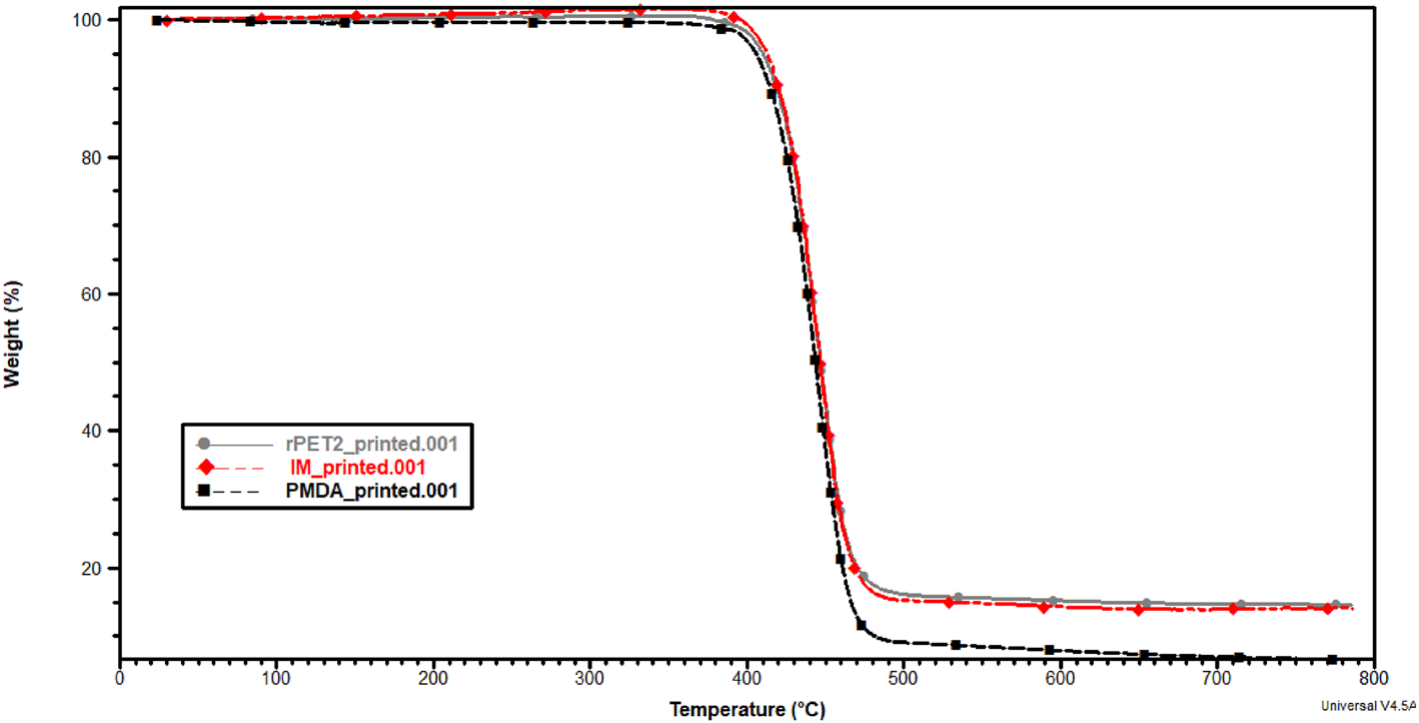
TGA curves for Printed rPET, rPET/PMDA and rPET/ IMs.

TGA thermographs are depicted in Figs. 6 and 7. Only one degradation peak around 420 °C for the filaments and printed samples is shown, indicating that the additives did not negatively impact the decomposition temperatures, especially for rPET/IMs compound that has high elastomeric contents. The single degradation peak also implies the homogeneity of the compounded rPETs and the uniform distribution of the additives within the rPET matrix. Furthermore, the degradation temperature of 400 °C guarantees that the rPET blends would not degrade during the extrusion temperature of 280 °C or the printing temperature of 260 °C.

### Mechanical properties

Uniaxial tensile, 3-point flexural, and Izod impact tests were carried out to reveal the effect of the additives on the mechanical properties. Tensile strength, modulus of elasticity, flexural strength, % strain at the break, and impact energy of plain rPET and compounded rPET were estimated for the printed samples.

#### Tensile tests

**Figure 8:**
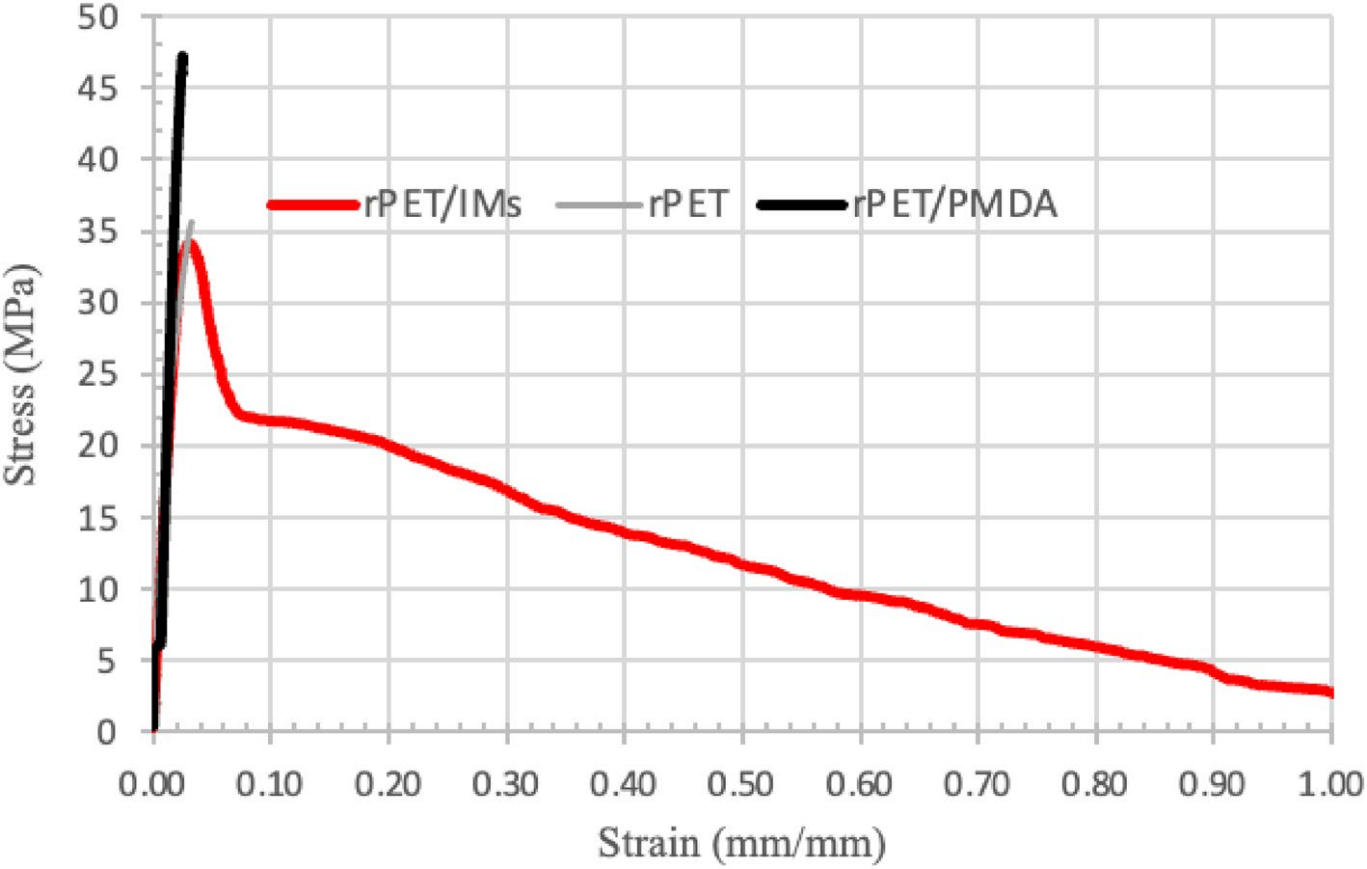
Representative tensile stress–strain curves for rPET, rPET/PMDA, and rPET/IMs.

Figure 8 displays the representative tensile stress–strain curves.

**Figure 9:**
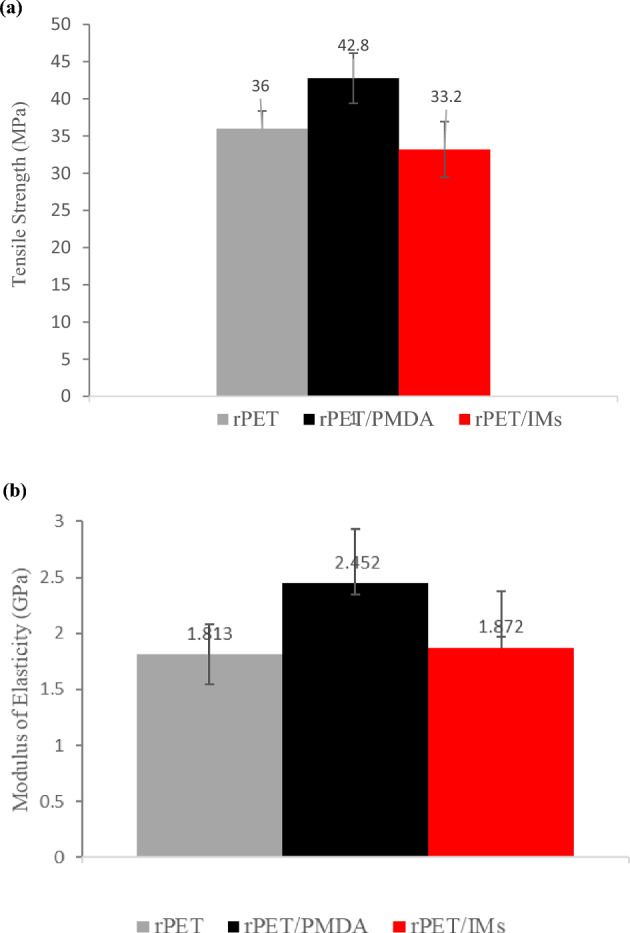
Average Tensile strengths (**a**) and Elastic moduli (**b**) for rPET, rPET/PMDA, and rPET/IMs.

Figure 9 is a graphic summary of the average tensile strengths and moduli of elasticity of the printed samples of plain rPET, rPET/ PMDA, and rPET/IMs. These figures revealed the positive impact of the additives on the strength, strain, and stiffness. Compared to the plain rPET, the rPET/IMs had a slightly lower average tensile strength and modulus of elasticity (approximately—6%); on the other hand, the rPET/PMDA showed an average increase of 25% in tensile strength and 30% increase in the elastic modulus. For the % elongation, as it was anticipated, the rPET/IMs showed the highest % elongation with an average of more than 100%, while the % elongation of the plain rPET and rPET/PMDA ranges from 3 to 6%. Similar results of the tensile strength, modulus of elasticity, and% elongation for the printed rPET (plain) were obtained by Zander et al.^35^; they reported tensile strength of 35 ± 8 MPa and 28 ± 9 MPa for the recycled and virgin PET samples, respectively and an average elastic modulus of 2112 ± 196 MPa. The % elongations were 2.5 and 3.5% for the virgin PET and rPET, respectively.

The average modulus of elasticity of rPET/PMDA increased substantially compared to the plain rPET. The improvement of the mechanical properties of rPET/PMDA printed samples agreed with the results obtained by Awaja et al. when different concentrations of PMDA were added to the recycled PET, but they used an injection molding process^28,29^. This might be attributed to the recombining of the broken PET chain through the chain extender, which increased the molar mass and chain entanglement, and, consequently, the stiffness. The branched structure is generated from the reactions between the end hydroxyl group of rPET and the PMDA. Asensio et al.^24^ attributed the increase in tensile properties to the aromatic groups in the chain extender.

A positive correlation between the crystallinity and the strength and stiffness of the rPET compounds was observed. The rPET/PMDA printed samples with the highest crystallinity of 14.3% recorded the highest tensile strength, tensile modulus, and flexural strength. Voorde et al.^36^ reported similar outcomes when they altered the degree of the crystallinity of the printed rPET by varying the cooling rate of the printing; the slower the cooling rate, the higher the crystallinity resulting in a higher modulus of elasticity.

When the impact modifiers were added with the chain extender (rPET/IMs samples), there was a slight decrease in elasticity modulus and tensile strength. The stiffness reduction could be attributed to the chemical interaction between the PET chain and the glycidyl methacrylate group of the reactive impact modifier (Lotader AX 8900)^42^. On the other hand, a substantial increase in the ductility measured by % elongation was noted. This was the anticipated impact of dispersing a soft rubbery phase within the rPET matrix to improve the impact strength, indicating that a high fraction of the reactive IM could be grafted into the rPET matrix, and uniform dispersion was achieved, as is shown in the SEM micrographs in Fig. 12.

#### Flexural tests

**Figure 10:**
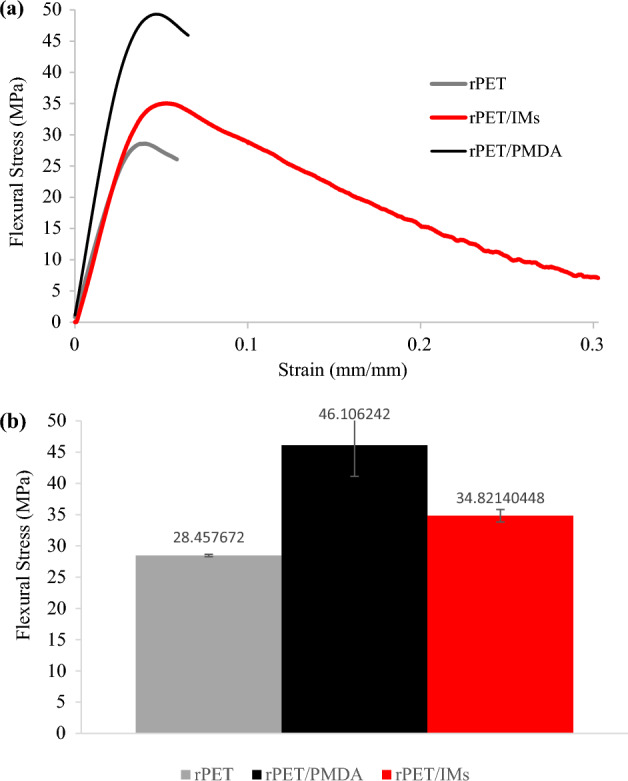
(**a**) Representative flexural stress–strain curves and (**b**) Average flexural strengths for rPET, rPET/PMDA, and rPET/IMs.

The three-point flexural tests were performed on the 3D printed samples according to ASTM D966 using MTS Insight 30 load frame with a 5kN load cell with the 3- point bending fixtures. Figure 10 shows the representative flexural stress–strain curves and the average flexural strength for rPET, rPET/PMDA and rPET/ IMs printed standard samples. The rPET /PMDA printed samples recorded the highest average flexural strength of 46 MPa, then rPET/IMs recorded 35 MPa, then the plain rPET recorded 30 MPa. The rPET/IM had the highest % strain at a break of 30%, then the plain rPET and rPET/PMDA at 6% elongation. This behavior is very similar to the tensile loading behavior.

#### Izod impact tests

**Figure 11:**
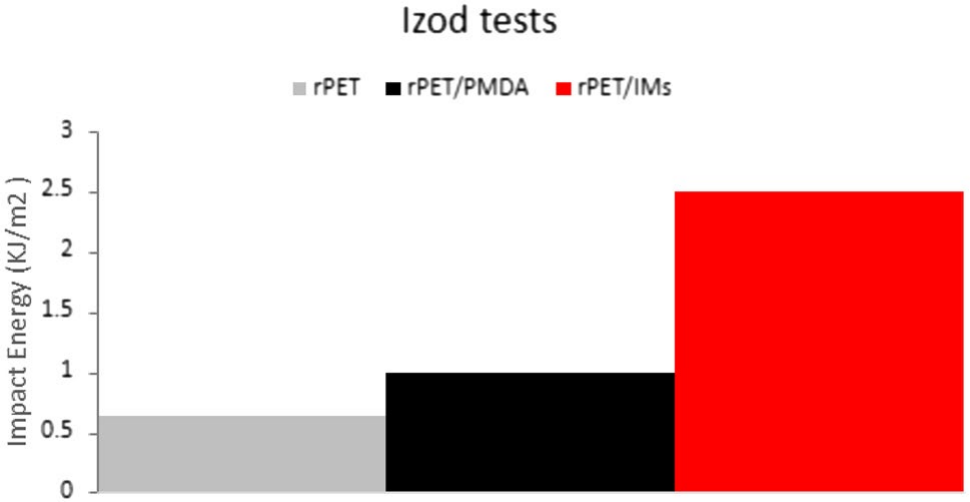
IZOD test for rPET, rPET/PMDA, and rPET/IMs.

**Figure 12:**
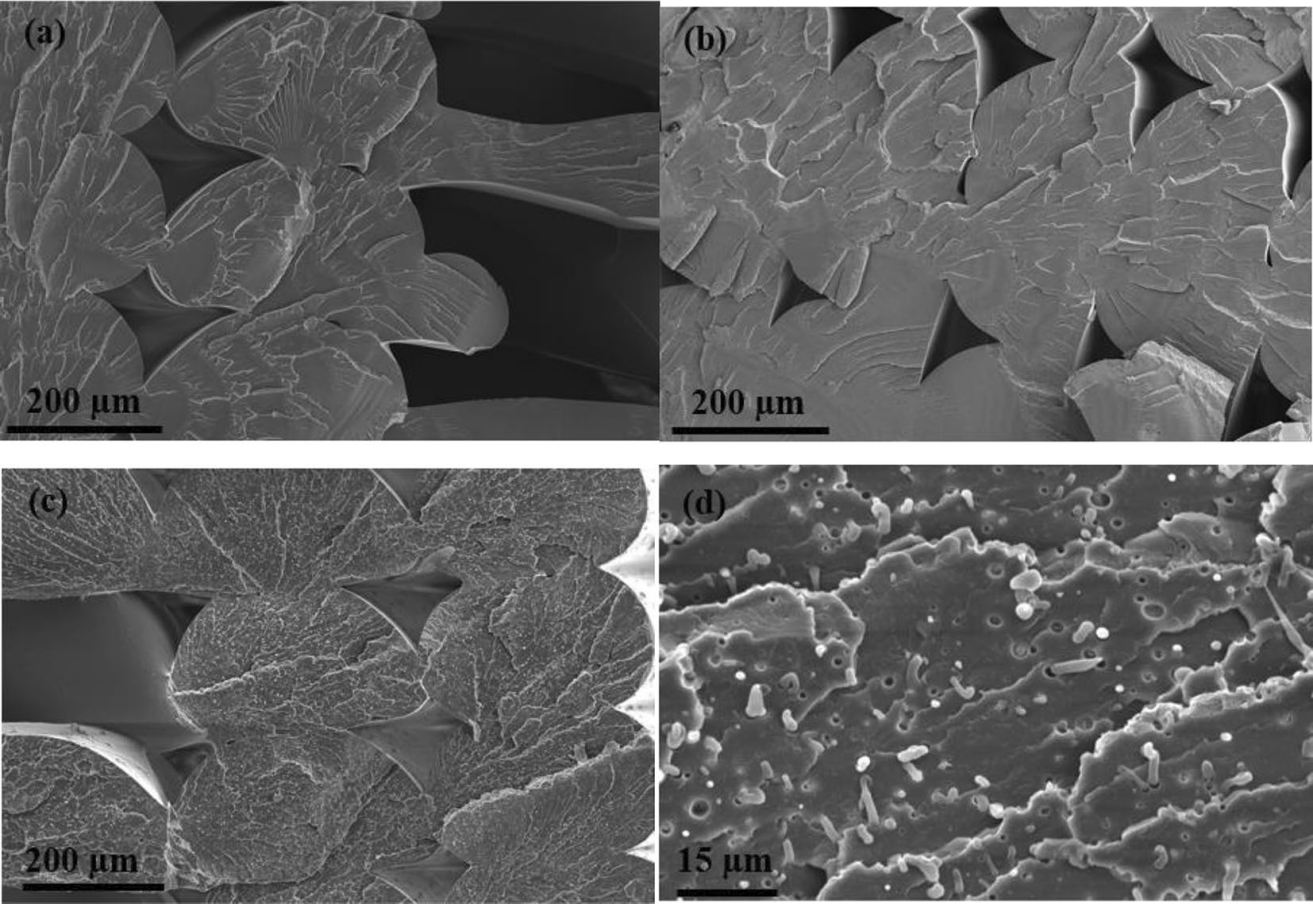
Fractured surfaces of (**a**) rPET, (**b**) rPET/PMDA, (**c**) rPET/IMs and (**d**) a high magnification micrograph of rPET/IMs showing the dispersed elastomeric impact modifiers.

The IZOD impact printed samples were tested according to the ASTM D256. Figure 11 shows that the compounded rPET /IMs printed samples have the highest impact energy. This enhancement in the impact energy indicates the well-dispersed phase of the reactive and non-reactive rubbery additives by grafting to the rPET matrix. The addition of the functionalized SEBS and lotader would prevent coalescence of the elastomeric particles and ensure proper interfacial adhesion of the rubbery additives into the matrix and forms a small uniform elastomeric well-dispersed phase^14^. The fractured surfaces of the rPET/IMs depicted in Fig. 12d also confirmed the well-dispersed phase of the reactive, non-reactive impact modifiers and SEBS within the rPET matrix.

Even though the reported values of the mechanical properties of printed rPET were lower than those of the injection molded samples, the mechanical properties could be improved further if the printing parameters were optimized. Suggestions to improve the mechanical properties include using a raster angle of 0°, reducing the printing speed, and increasing the printing bed temperature well above the T_g_.

### Fracture analysis

Morphological characteristics in fractograms of materials that have experienced a failure usually provide narrative features that connect to the failure process^43^. These include failure types, such as brittle or ductile, and failure modes, including corrosion, erosion, fatigue, and overload. In addition, the fractograms can also provide feedback regarding the filament properties and printing parameters. For these reasons, a fractography analysis of the fractured tensile surfaces was conducted to identify the failure mechanisms for the printed rPET variants. Figure 12a–c displays the electron micrographs of the fractured surfaces for rPET, rPET/PMDA, and rPET/IMs printed tensile samples, respectively. The failure types for the rPET and rPET/PMDA tensile samples, shown in Fig. 12a,b, display characteristics that indicate a brittle failure mode, failing suddenly with relatively small amounts of deformation. Moreover, the fracture surfaces displayed are almost flat and do not present areas of significant plastic deformation. On the other hand, the fractured surface of rPET/IMs, Fig. 12c, shows a degree of plastic deformation and a rougher fracture due to the dispersed elastomeric phase.

The most noticeable morphological difference in these fractograms is the impact modifiers, which can be observed as tiny fibers in the rPET/IM samples cross-section, Fig. 12d. The impact modifiers are uniformly dispersed in the rPET matrix, verifying that the parameters selected for filament extrusion produce a homogenous material. Additionally, when the rPET/IMs sample fails, the elastomeric impact modifiers undergo additional deformation, ultimately tearing and resulting in a pull-out effect on either end of the sample, producing cavities on the fractured surface. The mechanical response of the rPET/IMs samples confirms this behavior, as shown in Fig. 8. The stress–strain curves show a sudden decrease in stress around 35 MPa, followed by substantial elongation.

It is also worth noting that the size and occurrence of voids in the cross-section of the rPET/PMDA and rPET/IM samples are relatively low compared to plain rPET. This suggests that the compounded feedstock rPET has a high degree of fusion with surrounding material during deposition due to the existence of the additives. The presence of voids between deposited layers (inter-bead void) is inherent to the material extrusion printing process^44^. However, no intera-bead voids exist within the filament, suggesting the feedstock rPET materials are homogenous with no internal defects, and the reported twin-screw extrusion parameters are optimal.

## Conclusion

This paper focuses on extruding compounded rPET pellets with a chain extender, toughening agent, and impact modifiers into filament for MEX-AM technology to embrace the concept of circular economy and sustainable materials. Being able to customize the mechanical properties of the filaments with the utilization of low-cost additives without negatively affecting the thermal stability or requiring more processing steps is advantageous. Additionally, this approach provides a viable and sustainable feedstock for MEX-AM’s fast-growing industry/market.

Despite the defects in the printed samples, our experiments proved substantial improvement in the mechanical properties of the 3D printed samples when the rPET pellets were compounded with different additives. Our results showed that the melt flow index has improved by adding the chain extenders and the impact modifiers, which increases the melt strength during the multiple extrusion cycles. Furthermore, the mechanical properties were improved; this includes stiffness, tensile strength, impact energy, and flexural strength. The thermal behavior has not been significantly affected, which indicates good thermal stability even when elastomeric additives were utilized.

The fracture analysis of the printed samples showed a brittle fracture mode, which could be attributed to the printing process but not the filaments. The future work will involve optimizing the printing parameters to avoid defects in the printed parts. Some recommendations stemming from some trials include increasing the nozzle temperature, decreasing the printing speed, and increasing the printing bed temperature.

The positive outcomes obtained from this study will encourage researchers to investigate greener options in making feedstocks for fast-growing additive manufacturing technologies to mitigate the negative environmental impacts.

## Data Availability

The dataset generated and/or analyzed during the current study will be made available by the corresponding author on reasonable request.
